# Is Post-Operative Follow Up Affected by Stent Omission or Stent on a string after Ureteroscopy?

**DOI:** 10.51894/001c.144121

**Published:** 2025-09-30

**Authors:** Brandon Tompkins

**Affiliations:** 1 Department of Urology UM Health Sparrow

## 38

### INTRODUCTION

Although there has been an increased push for leaving patients stentless or leaving a stent on a string for self removal following ureteroscopy; the downstream effects on postoperative follow up are not well established. Postoperative follow up is important following ureteroscopy to evaluate for residual hydronephrosis and stone surveillance with imaging four to six weeks after surgery.

### OBJECTIVES

The primary objective of this study was to evaluate postoperative follow up when evaluating patients left stentless and or stent on a string vs having a stent placed after ureteroscopy.

### METHODS

This was a single center retrospective chart review, which consisted of one hundred and fifty eight patients between December 2018 to March 2020. The primary endpoint was to evaluate for postoperative follow up after ureteroscopy between those left stentless or with stent on a string for self removal vs having a stent placed.

### RESULTS

One hundred and fifty eight unique patient charts were reviewed following ureteroscopy. One hundred and eighteen patients were stented postoperatively, twenty four were left without a stent and sixteen were left with a stent on a string for self removal. Ninety eight (98%) of patients follow up in the clinic after their procedure when a stent was placed, but only sixty six percent (66%) when left stentless and fifty six percent (56%) when a stent was left on string for self removal. Patients either left stentless or with a string vs having a stent placed, were less likely to follow up outpatient after their surgery (P <0.05).

### DISCUSSION

Patients left stentless or with a stent on string for self removal are less likely to follow up postoperatively. Further studies evaluating the downstream effects of decreased postoperative follow up warrant further investigation. Additionally, new approaches to encourage postoperative follow up and adherence to completing postoperative imaging need further investigation.

